# Dietary Patterns and Components in Nonalcoholic Fatty Liver Disease (NAFLD): What Key Messages Can Health Care Providers Offer?

**DOI:** 10.3390/nu11122878

**Published:** 2019-11-26

**Authors:** Kiarash Riazi, Maitreyi Raman, Lorian Taylor, Mark G. Swain, Abdel Aziz Shaheen

**Affiliations:** 1 Department of Medicine, Division of Gastroenterology and Hepatology, University of Calgary, Calgary, AB T2N 4Z6, Canada; kriazi@ucalgary.ca (K.R.); mkothand@ucalgary.ca (M.R.); lorian.taylor@ucalgary.ca (L.T.); swain@ucalgary.ca (M.G.S.); 2 Community Health Sciences, O’Brien Institute for Public Health, Cumming School of Medicine, University of Calgary, 3280 Hospital Drive NW, Calgary, AB T2N 4Z6, Canada

**Keywords:** non-alcoholic fatty liver disease, Mediterranean diet, lifestyle change, weight loss

## Abstract

Nonalcoholic fatty liver disease (NAFLD) is a rising epidemic worldwide and will be the leading cause of cirrhosis, hepatocellular carcinoma, and liver transplant within the next decade. NAFLD is considered as the hepatic manifestation of metabolic syndrome. Behaviors, such as a sedentary lifestyle and consuming a Western diet, have led to substantial challenges in managing NAFLD patients. With no curative pharmaceutical therapies, lifestyle modifications, including dietary changes and exercise, that ultimately lead to weight loss remain the only effective therapy for NAFLD. Multiple diets, including low-carbohydrate, low-fat, Dietary Approaches to Stop Hypertension (DASH), and Mediterranean (MD) diets, have been evaluated. NAFLD patients have shown better outcomes with a modified diet, such as the MD diet, where patients are encouraged to increase the consumption of fruits and vegetables, whole grains, and olive oil. It is increasingly clear that a personalized approach to managing NAFLD patients, based on their preferences and needs, should be implemented. In our review, we cover NAFLD management, with a specific focus on dietary patterns and their components. We emphasize the successful approaches highlighted in recent studies to provide recommendations that health care providers could apply in managing their NAFLD patients.

## 1. Introduction

### 1.1. NAFLD Background

Deposition of excessive fat inside the liver, also known as fatty liver, can occur as a consequence of many factors, some of which include excessive alcohol consumption, viral infections, medications, and may be observed with other medical diagnoses such as inflammatory bowel disease [1,2]. However, when fatty liver is a consequence of over-nutrition together with insulin resistance, the condition is referred to as nonalcoholic fatty liver disease (NAFLD) [3]. NAFLD comprises a spectrum of liver histopathology ranging from the presence of a benign fatty liver, referred to as nonalcoholic fatty liver (NAFL), to fatty liver superimposed by inflammation, hepatocyte death, and fibrosis, also known as nonalcoholic steatohepatitis (NASH) [1]. NAFLD is the most common liver disorder worldwide [4]. Importantly, as recently reported, NASH is the second most common indication for being listed for liver transplant in the United States [5]. Most patients with NAFLD are either asymptomatic or experience non-specific symptoms, including fatigue and right upper quadrant abdominal discomfort. The diagnosis of NAFLD is frequently made incidentally following lab work identifying abnormal liver biochemistry, in the absence of any alternative explanation. Occasionally, the diagnosis may be suspected following hepatomegaly identification on physical examination, or sonographic features of steatosis [6]. Therefore, the subtle presentation, high prevalence, and the hepatic and extra-hepatic detrimental outcomes associated with NAFLD make it a serious global health problem. While obesity and overweight are established risk factors for NAFLD and weight loss is recognized as effective therapy, long-term sustainability of weight loss is achievable only in a limited proportion of patients [1]. The objectives of this manuscript are to present an overview of the prevalence, risk factors, and pathophysiology of NAFLD, and focus on a detailed review of dietary patterns as a NAFLD treatment independent of weight loss and provide key dietary messages for health care providers to implement into clinical practice. 

### 1.2. Natural History of NonAlcoholic Fatty Liver Disease

Under normal circumstances, there is only a small amount of fat deposit stored in hepatocytes [7]. NAFL is diagnosed when there is evidence, either by imaging or histology, that over 5% of hepatocytes are filled with fat, in the absence of any history of excessive alcohol use or any secondary causes of fatty liver [1]. The amount of alcohol is considered excessive when the patient reports drinking three units or more of alcohol per day for men and two units or more of alcohol per day for women [2]. Each unit or standard drink is equivalent to 10 g of alcohol, and is equal to 250 mL of beer, 200 mL of wine, or 30 mL of whiskey [2]. Interestingly, the definition of a standard unit varies in different countries [8]. The American Association for the Study of Liver Diseases has defined significant alcohol consumption as > 21 standard drinks per week for men and > 14 standard drinks per week for women [1]. In contrast, NASH is a pathological diagnosis, defined as the presence of NAFL complicated by inflammation and hepatocyte injury (e.g., ballooned hepatocytes or Mallory hyaline), with or without fibrosis [1]. NASH may be a progressive diagnosis, resulting in advanced stages of fibrosis or cirrhosis [1]. Aside from cirrhosis and the detrimental complications associated with it, NAFLD and NASH are concerning diagnoses due to the increased prevalence of hepatocellular carcinoma (HCC), especially with NASH [9,10]. The annual incidence of HCC in NAFLD patients has been estimated to be 0.44 per 1000 person/years, whereas in patients with NASH this incidence rate increases to 5.29 per 1000 person/years [4]. 

A recent meta-analysis of 86 studies from 22 countries with a total sample size of 8.5 million estimated the worldwide prevalence of NAFLD as 25.2% (95%CI: 22.1–28.7%) [4]. When stratified by geographical regions, the Middle East had the highest prevalence with 31.8% followed closely by South America with 30.5%, Asia with 27.4%, Europe with 23.7%, and North America with 21.1%. The lowest prevalence was reported in Africa with 13.5% [4]. The gold standard to diagnose NASH is a liver biopsy, an invasive procedure with possible major complications, limiting a population estimate. The meta-analysis identified the prevalence of NASH to be 59.1% (47.6–69.7) among biopsied NAFLD patients and 6.7% (2.2%, 18.7%) among patients biopsied for reasons other than NAFLD [4]. However, the global prevalence of NASH is estimated to be between 3% and 6% [11], while the prevalence of NASH cirrhosis has been estimated to be 0.18% [12]. The overall annual fibrosis progression rate from a baseline stage of no fibrosis (i.e., F0) in patients with NASH is estimated to be 0.09 stage (95% CI, 0.06–0.12), with 40.8% of patients showing fibrosis progression [4]. 

NAFLD is considered to be a component or hepatic manifestation of metabolic syndrome (MetS) [3]. MetS is a cluster of metabolic abnormalities that result from insulin resistance and are often identified in obese and sedentary patients [13,14]. MetS is diagnosed when central obesity in addition to any two of the following components are present in an individual: High blood triglyceride (TG) levels, low high-density lipoprotein (HDL) cholesterol levels, hypertension, increased fasting blood glucose, or established type 2 diabetes mellitus (T2DM) [15]. In Western countries, the prevalence of MetS is estimated to affect 20% of the adult population [16] and an increased prevalence of NAFLD has been reported in patients with MetS. For example, a meta-analysis of 80 studies from 20 countries in individuals with T2DM (*n =* 49,419) demonstrated a prevalence of NAFLD of 55.5% (47.3–63.7%) [17]. Similarly, MetS or T2DM has been shown to be present in as high as 85% of NAFLD patients [18]. Hypertension is also more common among NAFLD patients compared with a control non-NAFLD group (OR  =  1.24, 95% CI: 1.14–1.36) [19]. Since the components of MetS are all risk factors for cardiovascular morbidity and mortality, cardiac-related comorbidity and death are increased in patients with NAFLD. Of major concern, cardiovascular disease is the main cause of mortality in patients with NAFLD [1], significantly surpassing liver-related mortality among NAFLD patients (48% vs. 7%, respectively) [20]. Notably, only 0.1% of all deaths in the general population are liver related [20]. Although obesity and MetS are the major risk factors for NAFLD, up to 30% of NAFLD patients are neither obese nor have any of the MetS components [21]. Lean NAFLD cases highlight a role for genetic susceptibility in the pathogenesis of NAFLD [21].

### 1.3. Pathogenesis of NAFLD and NASH

The pathophysiological hallmark of NAFLD is insulin resistance (IR) [22]. IR leads to elevated portal and circulating free fatty acids, which subsequently result in increased hepatic uptake. When increased hepatic fatty acid input (uptake and biosynthesis) overcomes hepatic clearance by beta-oxidation, or by secretion as very-low-density lipoprotein (VLDL), fatty acids accumulate within hepatocytes as triglycerides (TGs) [23,24,25]. These TGs are secreted into the bloodstream in the form of VLDL to be transferred to adipose tissues. Under conditions where hepatocytes are unable to fully secrete all TGs as VLDL, the TGs are stored as cytosolic lipid droplets within hepatocytes as lipid vesicles isolated from the cytoplasm by a phospholipid monolayer [26].

The source of fatty acid (FA) input to the liver is through either dietary fat intake, in the form of chylomicrons or plasma-free FA pool (also known as circulating non-esterified fatty acids (NEFAs), which are released from adipose tissue) [23]. Additionally, the hepatic uptake of excess dietary carbohydrates can also be used as a substrate for FA biosynthesis, and is another important source for FA input to the liver [24]. The relative contribution of each source of FA is different between fed and fasting states. Donnelly et al. (2005) showed that in the fasting state, the contributions of peripheral NEFAs, FA biosynthesis, and dietary fats to hepatic fat content were 59% (45.1–74.3%), 26.1% (12.7–37%), and 14.9% (4.3–28%), respectively [24]. Therefore, NEFA originating from visceral adipose tissue may be an important lipid source for increased hepatic fat content in NAFLD, followed by de novo biosynthesis and diet. Free fatty acids (FFAs) may be oxidized for routine use; however, in NAFLD, increased FFA oxidation causes oxidative stress that uncouples mitochondrial oxidation and phosphorylation and generates reactive oxygen species (ROS) [25]. Mitochondria have several defense mechanisms against toxic by-products, such as ROS (e.g., anti-oxidants, glutathione, catalase, etc.); however, if these defense mechanisms are overcome, organelle damage may occur [27]. Mitochondrial damage might result in decreased lipid oxidation or even cell death, which in turn can lead to inflammation, production of cytokines, recruitment of inflammatory cells, generation of fibrosis, and progression to NASH [25].

Although the pathophysiological outcomes of NASH progression are not completely understood, it has been proposed that in NAFLD, the liver is vulnerable to parallel hits stemming from localized oxidative stress and pro-inflammatory mediators generated in peripheral tissues [28]. The literature related to the gut–liver axis and its relationship to NAFLD is growing, as multiple preclinical and clinical studies have identified an increased prevalence of small intestinal bacterial overgrowth, and changes in gut microbial composition and their influence of intestinal permeability in NAFLD/NASH [29,30]. Advanced microbiome assessment methods, such as shotgun metagenomic sequencing, have identified an association between gut microbiome signatures and the presence of advanced NAFLD fibrosis [31]. Furthermore, gut microbiome dysbiosis may contribute toward intestinal barrier damage, leading to hepatic inflammation in the setting of enriched dietary fat composition [32]. High fat diets and diets depleted in fiber lead to reduced thickness of the intestinal mucous layer, redistribution of tight junction proteins, and low-grade inflammation. Moreover, bacterial components and metabolites derived from actions of the gut microbiome on dietary, environmental, and bile acid exposures can reach the portal vein and promote inflammation, further supporting targeting dietary composition as a therapeutic opportunity in NAFLD management [33].

### 1.4. Risk Factors for NAFLD

The non-modifiable risk factors for NAFLD include age, sex, genetic background, ethnicity, and family history of fatty liver, T2DM, or premature cardiovascular disease [34]. In general, the prevalence of NAFLD- and NAFLD-related fibrosis increases with age [35]. For adults under 50, NAFLD is more prevalent among men [36]. However, the prevalence of NAFLD in postmenopausal females is similar to males after the age of 50 [36]. Several gene variants predispose to NAFLD by enhancing fat accumulation in the liver, the most important of which is human patatin-like phospholipase domain-containing 3 (PNPLA3). PNPLA3 is a key regulator of lipid droplet turnover in hepatocytes and hepatic stellate cells [37]. PNPLA3 rs738409 is a single nucleotide substitution of cytosine to guanine, which encodes for a nonsynonymous change, resulting in the substitution of isoleucine to methionine at position 148 (I148M) of the protein [38]. This sequence variation is believed to be the strongest genetic determinant of the full spectrum of nonalcoholic fatty liver disease, from fatty liver formation to NASH and HCC [37,38,39,40].

Obesity, over-nutrition, dietary composition, and inactivity are important modifiable risk factors for NAFLD. Obesity, especially central obesity, is possibly the most important modifiable risk factor for NAFLD, and is a result of over-nutrition and inactivity [34]. The majority of patients with NAFLD are either obese or overweight. The overall prevalence of obesity among NAFLD patients worldwide has been estimated at 51.3%, and 81.8% of NASH patients are obese [4]. Results from 12,454 adult participants in the Third National Health and Nutrition Examination Survey (NHANES) describe the prevalence of NAFLD in various Body Mass Index (BMI) categories: 7.5% in normal-weight men (BMI of 18.5–24.9), 38.6% in obesity class 1 (BMI of 30.0–34.9), and 56.6% in obesity class 2 (BMI 35 or above) [41]. Similar trends have also been reported for women with NAFLD: Prevalence of NAFLD was 6.7% in women with a normal BMI compared to 24.7% in women with a BMI of 30.0 to 34.9, and 44.3% in those with a BMI of 35 and above [41]. Central obesity, which is measured through waist circumference (≥ 102 cm for men and ≥ 88 cm for women), is an independent risk factor for both NAFLD and MetS [42]. It is a widely accepted hypothesis that visceral adipocytes are detrimental to the liver as they release more fatty acids, pro-inflammatory cytokines, and adipokines than peripheral adipose tissue pools [3]. 

Modern societies spend increasingly more time being sedentary, and less time engaged in physical activity, as a result of environments that minimize activity and require prolonged sitting times at work, in the home, and for transportation [43]. Sedentary behavior is defined as low-energy expenditure in a sitting or reclining position during waking hours, and it has been linked to chronic low-grade inflammation and contributes to obesity [44]. High sitting times (e.g., >8 h/day) compared to low sitting times (e.g., < 4 h/d) almost double the risk of developing type 2 diabetes and increase the incidence and mortality risk associated with cancer and CVD by 10% to 20% [45]. The association between sedentary lifestyle and obesity, MetS, and T2DM has been supported by epidemiological research [44,46,47]. Low levels of moderate-intensity physical activity and high amounts of sedentary time are associated with insulin resistance, T2DM [48], and NAFLD [49,50]. 

The relationship between diet and the development of NAFLD is complex and extends beyond total energy intake. Certainly, over-nutrition results in obesity by altering the energy balance. However, dietary patterns may have an independent effect on NAFLD apart from energy density. A “Western” style diet is a well-recognized diet pattern associated with MetS and NAFLD [51,52,53]. This pattern is generally hypercaloric; consists of high intakes of animal products high in saturated fat, refined sugars, and grains; sugar-containing soft drinks; and high intakes of processed food high in trans-fats and low in fiber and phytochemicals [54]. The high glycemic index associated with the Western dietary pattern results in a rapid increase in insulin and postprandial serum glucose levels and contributes to the induction of liver lipogenesis and VDRL secretion, which can result in obesity [54,55]. In addition to the Western dietary pattern, consumption of restaurant-based fast-foods are another example of an energy-dense, low-nutrient rich diet [56]. In a clinical trial in Sweden, 18 healthy volunteers were given at least two fast-food-based meals a day with the goal of doubling the regular caloric intake, in combination with adoption of a sedentary lifestyle for 4 weeks, and were compared to a control group [55]. The results showed an average 6.4-kg weight gain, a significant rise in serum alanine aminotransferase (ALT) levels, and an increase in average intrahepatic triglyceride levels from 1.1% to 2.8% compared to controls [55]. Of note, eating patterns have also been associated with NAFLD. A prospective study of 2,254 NAFLD-free subjects over three years found that individuals who habitually eat before bedtime had double the risk of developing NAFLD [57]. Hyper-caloric snacking between meals results in increased liver fat; however, consuming these same snacks with meals did not cause liver fat accumulation [58]. 

### 1.5. NAFLD Management Overview

The management of NAFLD has focused on reducing insulin resistance, limiting oxidative stress, and modifying underlying risk factors [59,60]. Lifestyle modification remains the most effective treatment for NAFLD and NASH, and is achieved through a combination of dietary modifications and physical activity. Nevertheless, there are several challenges to achieving these outcomes [61,62,63,64]. Aggressive weight loss can aggravate liver inflammation in subjects with NAFLD, if it reaches a rate greater than 1.6 kg per week [65]. Furthermore, weight loss maintenance is challenging, limiting long-term results. This weight gain is attributed to the challenges of maintaining a hypocaloric diet in an environment of overabundance and easy access high-energy, palatable food options. Consequently, it is important to define which dietary components are most likely to induce NAFLD, allowing for the targeted increase in availability of resources and programs to assist patients with long-term weight loss maintenance and achieving success. In NAFLD patients with more advanced liver disease stages, and in those patients with high genetic risk or in the presence of diabetes, pharmacological treatment might be necessary to intensify lifestyle interventions, although for the time being there is no approved drug for the treatment of NAFLD. 

### 1.6. Lifestyle Modification in NAFLD

Weight loss in NAFLD is effective in improving liver disease severity. A recent meta-analysis evaluated the effect of weight loss on NAFLD-related biomarkers from 22 clinical trials (2588 combined subjects) using a variety of interventions [66]. These interventions included behavioral weight loss programs (15 studies), pharmacological (6 studies), and surgical procedures (1 study). Results were compared to a lower-intensity weight loss intervention. In patients with NAFLD, weight loss (median −3.61 kg) achieved within the 6-month program was associated with significant improvements in ALT levels, liver steatosis, histologic NAFLD activity score, liver stiffness, and the disappearance of NASH [66]. However, there was no significant effect on liver fibrosis score [66].

Most studies relating to the association of weight loss with NAFLD have focused exclusively on NASH, possibly due to the importance of NASH in the development of adverse clinical outcomes, such as cirrhosis. For example, a randomized controlled trial by Wong et al. (2013) in a group of 154 NAFLD patients compared the effect of a dietitian-led lifestyle modification program for 12 months linked to a standard care protocol that included physician follow-up on remission of NAFLD. Remission was defined as a reduction in intrahepatic TG content to less than 5%. Findings indicated that a 10% weight reduction lead to remission of NAFLD in 97% of the patients, compared to only 13% in patients who lost less than 3% body weight [67]. Another study examining the effect of the drug orlistat in NASH patients found that at least a 5% weight reduction was required to improve the NASH activity score (NAS), liver fat content as well as insulin sensitivity, when compared to those who did not lose weight [68]. Vilar-Gomez and colleagues studied the effect of a 52-week weight loss program in 293 patients on histological features of NASH [69]. They compared pre- and post-liver biopsy results and demonstrated that the degree of weight loss was independently associated with improvements in all NASH-related histological parameters. In their study, 90% of patients who lost ≥ 10% of body weight had a resolution of NASH, and 45% had liver fibrosis regression; however, only 10% of study participants were able achieve a 10% weight loss [69]. The beneficial effects of weight loss on NASH-related liver parameters were still significant even with a 5% to 10% weight loss, and 30% of participants were able to achieve this degree of weight loss [69]. 

### 1.7. The Effect of Exercise on NAFLD

Physical activity has numerous beneficial effects on MetS. Increasing physical activity reduces intrahepatic triglyceride content and markers of hepatocellular injury in patients with NAFLD, independent of weight loss [70]. A systematic review and meta-analysis from 17 studies on the impact of structured exercise training, and associated weight loss, on intrahepatic triglycerides (IHTGs) in individuals with NAFLD, showed exercise reduced IHTG levels independent of significant weight change. However, the benefits achieved were substantially greater when weight loss occurred [71]. The guidelines from the European Associations for the Study of the Liver (EASL), Diabetes (EASD), and Obesity (EASO) recommend 150 to 200 min/week of moderate-intensity aerobic physical activity for NAFLD patients, in three to five sessions [72]. 

Since the majority of clinical trials in NAFLD have focused on the effects of a combined physical activity and diet therapeutic approach in NAFLD, studies focusing on physical activity alone are less common. In a physical activity-only systematic review of 28 randomized clinical trials in a combined total of 1644 participants, increased physical activity was associated with a significant reduction in liver fat content and serum aminotransferase levels, a significant decrease in BMI, and improved peripheral insulin sensitivity [73]. The effect of physical activity on hepatic liver fat content was more prominent in young patients and patients with a higher baseline BMI [73]. In line with these findings, a clinical trial by van der Heijden [74] also showed that a 12-week controlled moderate-intensity physical activity program without weight loss resulted in a significant decrease in hepatic fat content and visceral fat content. However, insulin resistance decreased only in obese adolescents and not in lean adolescents. Considering the difficulty obese patients have in losing weight on a hypocaloric diet, and the independent benefits of physical activity, setting realistic goals for increasing moderate-intensity activity provides another option for the patient with NAFLD. 

In regard to the optimal type of physical activity training (aerobic vs. resistance), a systematic review and meta-analysis of 12 randomized clinical trials (13 aerobic and 4 resistance exercise protocols) showed that both aerobic and resistance exercise improve hepatic steatosis [75], and also significantly improve NAFLD [70]. 

### 1.8. The Effect of Diet on NAFLD

Long-term adoption of a low-calorie diet is associated with decreased liver fat, and an improvement in cardiovascular risk [76]. Decreasing caloric intake by at least 30% (or by approximately 750 to 1000 kcal/day) resulted in an improvement in IR and fatty liver. However, as discussed previously, the sustainability of such hypocaloric diets is low. The multifactorial origin of NAFLD and NASH has led to management strategies that incorporate a combination of dietary interventions aimed at tackling the multiple hits occurring during the onset and evolution of this disease. For example, dietary patterns that reduce the intake of high glycemic index foods, increase dietary fiber and resistant starch, reduce saturated fat, and promote mono-unsaturated fatty acid (MUFA) and polyunsaturated fatty acid (PUFA) intake may have a positive effect on gut microbiota composition and function, intestinal barrier function, and IHTG accumulation.

Dietary patterns that leverage the above described principles may reduce the development of NASH, possibly even in the absence of weight loss. Here, we discuss dietary regimens recommended for the management of NAFLD patients. The four most common dietary patterns include the low-carbohydrate diet, the low-fat diet, the Dietary Approaches to Stop Hypertension (DASH) diet, and the Mediterranean diet.

High-carbohydrate diets (50% to 65% of calories from carbohydrates) have been associated with increased insulin resistance and obesity and shifting benign fatty liver toward NASH [77]. Low-carbohydrate diets are diets in which less than 40% of calories are from carbohydrates [78]. NAFLD patients receiving low-carbohydrate diets had a significant weight loss only in the short term (6 months) compared to those consuming a conventional high-carbohydrate/low-fat diet, but at one year, no difference in weight loss was observed [78]. Subjects on low-carbohydrate diets show improvements in biochemical parameters of MetS in comparison with those on high-carbohydrate diets. The ketogenic diet (8% carbohydrate, 30% protein, and 61% fat) is a severely carbohydrate-restricted diet and induces weight loss, decreases serum TG levels, and increases HDL levels compared to a high-carbohydrate diet (in the short term), especially in men [79,80]. However, adherence rates to low-carbohydrate diets in the long term are poor, and the potential effects of ketogenic diets on micronutrient deficiencies and the gut microbiome are unknown [81]. Adverse metabolic effects, such as thirst, fatigue, and even tachycardia, have been reported with the ketogenic diet [82].

The low-fat diet limits fat intake to 30%, with 50% of calories coming from carbohydrate and 20% from protein, and includes a restricted intake of trans-fat, saturated fat, and cholesterol (no more than 300 mg/day) [83,84]. Low-fat diets are intended to reduce the occurrence of conditions, such as heart disease and obesity [85]. In NAFLD, the comparison of low-fat and low-carbohydrate diets has demonstrated inconclusive results. For example, when prescribed to overweight people, both diets are equally effective for weight loss [86]. In contrast, one study in Korean NAFLD patients showed greater efficacy for a low-carbohydrate diet in reducing total energy intake and hepatic fat content [87]. The authors explained their findings based on low-fat energy consumption as part of the habitual diet, with less than 20% of calories from fat [87]. It is important to note that low-fat diets generally do not distinguish between the type of dietary fat consumed. All fats, whether saturated, monounsaturated, or polyunsaturated, are treated similarly, although the biological effects likely differ.

The DASH diet was first introduced for the management of hypertension and was designed to focus on lowering energy-dense food items and decrease total fat, saturated fat, and cholesterol. This dietary pattern is low in added sugars, sugar-sweetened beverages, and red and processed meats, and is enriched in vegetables, fruits, low-fat dairy products, whole grains, poultry, fish, and nuts [83]. It is rich in potassium, magnesium, and calcium, as well as protein and fiber [83]. The DASH dietary pattern is associated with a reduced risk of cardiovascular disease [88]. In a randomized controlled clinical trial including 60 overweight or obese adults with NAFLD diagnosed by ultrasonography, adherence to the DASH diet for 8 weeks, compared to a contemporary control diet, was significantly more effective in reducing weight, and improving liver enzymes, markers of insulin resistance, serum triglycerides, and VLDL levels [89]. A cross-sectional study in 3051 40 to 75-year-old subjects showed that adherence to the DASH diet was independently associated with a significantly lower prevalence of NAFLD [90]. The DASH diet has been demonstrated to improve blood pressure and hyperlipidemia, which may also translate to protective effects in NAFLD [91]. Nevertheless, despite benefits of the DASH diet for T2DM and cardiovascular disease [88], the evidence for its efficacy in NAFLD is limited. 

The Mediterranean (MD) diet has been recommended as the diet of choice for the treatment of NAFLD by various clinical practice guidelines [92], and has the greatest evidence to prevent and manage MetS and its components, particularly T2DM and CVD [93]. In the MD diet, high consumption of extra-virgin olive oil, vegetables (especially root and green varieties), fruits (particularly fresh), whole grains (cereals, breads, rice, or pasta), legumes, nuts (walnuts, hazelnuts, or almonds), and moderate consumption of fish (especially fatty fish rich in omega-3 fatty acids), low-fat dairy products, and red wine is recommended over the consumption of red and processed meat, processed and high-sugar food, refined carbohydrates and high-fat milk products [83]. The MD diet has a higher fat content (about 40% of total calories), is low in saturated fat (less than 10% of total calories), high in MUFA and omega-3 PUFA, with a decreased omega-6 PUFA content, and high in fiber (25 and 35 g/day for women and men, respectively) [83]. Strong evidence supports the benefits of the MD diet in controlling body weight, fasting plasma glucose, serum TG, and blood pressure [88]. Recently, Kaliora et al. studied the effect of the MD diet on clinical, biochemical, and inflammatory profiles in patients with simple NAFLD. Untreated NAFLD patients with no significant fibrosis (*n* = 44) underwent a 24-week diet intervention after nutritional counseling to increase adherence to the MD diet. Adherence to the diet was associated with significant improvements in liver imaging, liver fibrosis score, blood pressure, fasting glucose, hemoglobin A1c, and several other biomarkers, compared with pre-intervention values [94]. In another study, 278 participants with abdominal obesity/dyslipidemia were randomly assigned to either low-fat (LF) or Mediterranean/low-carbohydrate (MD/LC + 28 g walnuts/day) diet for 18-months [95]. The MD/LC diet induced a greater reduction in hepatic fat content compared to the LF diet, which reached statistical significance after 18 months (−4.2% vs. −3.8%, *p* = 0.04). Interestingly, the advantageous effect of the MD/LC diet in reducing hepatic fat content compared to that obtained with the LF diet was significant in both patients without NAFLD (hepatic fat content ≤ 5%, *p* = 0.04) and in NAFLD patients (hepatic fat content > 5%, *p* = 0.014). Compared to the LF diet, the MD/LC diet induced a greater increase in HDL and a greater decrease in blood pressure, triglycerides, and the triglyceride/HDL ratio [95].

### 1.9. Surgical and Pharmacological Treatment of NAFLD

Bariatric surgery has been used to control obesity and as a consequence, treat NAFLD. Significant improvement in the prevalence and severity of fatty liver occurs following bariatric surgery, although its benefits and safety in patients with NASH have yet to be established [96].

Since insulin resistance has been implicated in the pathogenesis of NAFLD, insulin-sensitizing agents have been investigated, with most studies examining the use of metformin and pioglitazone. Metformin is a biguanide drug that works through different mechanisms to lower blood glucose levels, including a reduction in postprandial and fasting glucose levels and the inhibition of hepatic and renal gluconeogenesis [97]. In contrast, pioglitazone is a thiazolidinedione that inhibits hepatic gluconeogenesis and enhances glucose uptake in muscles and adipose tissue through activation of peroxisome proliferator-activated receptor gamma (PPARγ) [59]. A systematic review and meta-analysis done on the efficacy of these two medications in NASH patients demonstrated that metformin (*n* = 81 patients) did not significantly improve liver histology scores for steatosis, ballooning, or fibrosis, while it significantly worsened lobular inflammation [98]. Pioglitazone, on the other hand, improved steatosis and lobular inflammation but did not improve fibrosis [98]. Considering the recognized role of inflammation and oxidative stress in the pathogenesis of NASH, vitamin E, an antioxidant, has been studied as a treatment option in NASH. Although the results indicated that while a daily dose of 800 to 1000 IU does not improve fibrosis, it does significantly improve histologic scores, lobular inflammation, and ballooning in non-diabetic adults with biopsy-proven NASH [98]. However, additional data is required to show that vitamin E is beneficial in other groups of NAFLD patients [76]. While many new drugs are being investigated in NASH clinical trials, these pharmacological treatments are currently limited to patients with biopsy-proven NASH in the context of clinical trials [1]. 

## 2. NAFLD Key Messages for Health Care Providers 

NAFLD is a chronic disease that is treatable through significant lifestyle modifications. Weight loss achieved through healthy diet and moderate-intensity physical activity remains the cornerstone for NAFLD management. We recommend the following key messages, in order of priority, that health care providers may consider when engaging patients in lifestyle change. 

### 2.1. Engage Patients in Their Management Plan 

While there is no widely accepted definition of patient engagement, the concept is that patients are actively involved in their own health. This activity may include reflection on how care fits into patient priorities and how patients choose to act on these decisions [99]. Engaged patients strive to be informed about their health, are involved in health care decisions, and participate in self-care, by assuming responsibility and accountability for the role their behaviors contribute in their health outcomes [100]. It is important to allow patients to choose and monitor lifestyle goals that they feel they are most likely able to accomplish. This focus will help build self-confidence and increase their chance of achieving successful behavior changes. A valuable lesson comes from the diabetes prevention program (DDP) clinical trial. In this large trial, 19% of participants were able to accomplish all three lifestyle change goals over one year: 7% or greater weight loss, increased physical activity to 150 min of moderate-intensity activity per week, and a reduction of total dietary fat to less than 25% of calories [101]. Interestingly, only14% of participants were not able to achieve any goal. The DDP demonstrated positive health outcomes as patients used a 1-year program that allowed participants to perform goal setting and work on behavioral changes progressively over time. While a program like the DPP may be necessary to support a 5% to 10% weight loss, health care providers can encourage positive dietary and physical activity changes to their patients with NAFLD. Health care providers can engage patient participation to lifestyle interventions and provide specific dietary and lifestyle advice as described below. However, suggesting weight loss in the absence of a concrete plan may disengage patients from their care and reduce chances of a successful outcome.

### 2.2. Utilize a Multidisciplinary Approach to NAFLD Management

To effectively support optimal health, health care systems need to create an environment in which the health-promoting choice is the easiest default option. Recent UK and European guidelines advise that NAFLD management should be multifaceted and patients should be managed using a multidisciplinary approach [72,102]. However, there is still a paucity of best practice data describing how such services should be shaped and delivered [103]. A multidisciplinary approach to the management of patients with NAFLD in an observational cohort study was associated with improvements in liver-related and cardio-metabolic-related health parameters with evidence of cost-effectiveness [103]. Care was delivered conjointly by hepatologists, diabetologists, and supported by specialist nurses, with an emphasis placed on weight management through lifestyle modifications. Another multidisciplinary clinic staffed by a hepatologist and diabetologist with dietetic support offered clinical dietary and lifestyle assessment and provided personalized lifestyle advice tailored to requirements [104]. Recommendations for exercise were also provided. Following 12 weeks, improvements were evident in ALT, weight, and total cholesterol. Hemoglobin A1c, TG, and systolic blood pressure improved in patients who had abnormalities at baseline [104]. Benefits for a multidisciplinary approach may include reinforcement of key messaging, intervention programming from clinical experts personalized to the individual patient (dietetic, exercise), establishment of accountability through long-term follow-up, and co-location of services to manage time and cost.

### 2.3. Encourage Patients to Start a Mediterranean Diet 

The Predimed study identified a number of dietary behaviors that were representative of an MD dietary pattern [105]. We propose that these dietary behaviors can be grouped into four categories and presented to patients with NAFLD to identify goal setting. 

Possibly, the most beneficial MD category for patients with NAFLD to prioritize as a goal is greater intake of fruits and vegetables. Fruits and vegetables are rich in dietary active compounds, including phytochemicals, antioxidant compounds, vitamins, phenolics, flavonoids, and dietary fibers, all of which have been shown to have health benefits in managing metabolic syndrome and its components [106,107,108]. To classify patients as eating more fruits and vegetables that are in line with an MD, they need to consume: (1) Four or more servings of vegetables a day, where two or more of these servings are consumed as raw salad greens. One serving is half a cup of fresh, frozen, or cooked vegetables, or one cup of green leafy vegetables. This recommendation is met, for example, if patients eat one cup of vegetables or two cups of salad; and (2) three or more servings of whole fruit or one and half cups of fruit a day. One serving equals half a cup of fresh or frozen fruit. An example of this would be eating an apple and banana for a midmorning and afternoon snack and half a cup of berries with breakfast or as an evening snack.

The second key dietary message would be decreasing high-sugar and/or high-fructose beverages and foods. It has been proposed that fat accumulation in the liver is linked to the metabolism of fructose. The MD provides much lower levels of added sugars and fructose than a typical Western dietary pattern [109]. To be consistent with an MD, a patient can: (1) Decrease the intake of commercially prepared or highly sweetened baked goods to less than two times a week. Examples of these include store-bought sweets, pastries, candy, and snacks like cookies, chocolate bars, doughnuts, and cakes; and (2) drink less than one can of sugar-sweetened beverage (355 mL or 12 oz) a day, recognizing that drinking no sugar-sweetened beverages is the best choice.

The third key dietary message is to modify the amount of added dietary fat, consuming primarily extra-virgin olive oil (EVOO). EVOO is a critical component of the MD [110], and has been shown to exert hepatoprotective effects through the induction of cellular antioxidant responses and inhibition of inflammatory pathways [111,112]. To increase olive oil (MUFA) and decrease added saturated and trans-fat in the diet, four behaviors are measured: (1) Using EVOO as the main fat in all cooking and baking; (2) consuming four or more tablespoons of EVOO a day depending on calorie requirements. A more realistic goal for weight management may be to aim for at least one tablespoon of EVOO a day; (3) eating three servings of nuts, seeds, or nut butters each week. One serving equals 30 g (1 oz); and (4) limiting consumption of butter, hard margarine, and cream to one tablespoon (15 mL) or less.

The fourth key dietary message is to choose meat and plant proteins that are higher in beneficial fats or fiber and lower in saturated fat. Reduced consumption of saturated fatty acids due to a low red meat content, and a high content of mono- or poly-unsaturated fatty acids, due to a healthy amount of EVOO, nuts, whole grains, and fatty fish has been shown to protect against NAFLD; possibly mediated partially through mechanisms involving the gut microbiome, intestinal barrier function, and gut–liver axis, as described earlier [108,113]. To follow an MD, patients should: (1) Choose white meats more often than red meats; (2) try to eat 300 to 450 g per week of fatty fish, such as salmon, herring, char, anchovies, sardines, or trout. This could be three 100- to 150-g servings a week; (3) eat three or more servings of beans, peas, lentils, or chickpeas each week. One serving is one half to two-thirds of a cup; and (4) eat less than one serving of red and processed meat a day. Some recommendations suggest eating less than two 75-g servings a week of red meat, and limiting processed meat consumption to a maximum of one serving a week.

The final key dietary message is to promote a healthy home-based eating environment where time is shared with family and friends. Promoting eating at home, with home-prepared foods, helps decrease the intake of fast food and processed meals. A recent cross-sectional study compared fast food consumption, including soft drink and sweet and salty snack intake; only high consumption of fast food compared to low consumption was associated with increased levels of alanine aminotransferase (OR: 2.74; 95% CI: 1.57–4.76) [114].

### 2.4. Educate Patients on the Importance of Physical Activity

We reviewed the association between sedentary lifestyle and the risk of developing NAFLD, MetS, and cardiovascular disease. Moderate-intensity physical activity requires a moderate amount of effort and noticeably accelerates the heart rate and breathing without being out of breath [115]. Examples include brisk walking, dancing, sports activities, resistance training, and household activities that increase the heart rate. Vigorous activities get the heart pounding and make breathing very fast. Examples include bicycling up a hill, fast swimming, and exercise classes [115]. We showed that moderate-intensity physical activity is associated with improved outcomes among NAFLD patients. The type of physical activity, whether aerobic or resistance training, could be recommended based on individual factors, including personal preference, level of fitness, and exercise tolerance [75]. Health care providers should recommend 30 to 45 min of moderate-intensity activity in three to five sessions a week for a total of 150 to 200 min [72]. Referring all NAFLD patients to community support is relatively easy to do and appears to increase the likelihood of improving patients’ activity levels [116].

Finally, success in a lifestyle change relies on mutual understanding from patients and health care providers that it is a long-term process that should be personalized to meet the patient’s needs and preferences. Health care providers should provide patients with adequate resources to empower them to succeed in this journey, and conversely, patients should demonstrate accountability to their health care team by presenting for scheduled follow-ups to discuss facilitators and barriers to outcomes.

## References

[1] Chalasani N., Younossi Z., Lavine J.E., Charlton M., Cusi K., Rinella M., Harrison S.A., Brunt E.M., Sanyal A.J. (2018). The diagnosis and management of nonalcoholic fatty liver disease: Practice guidance from the American Association for the Study of Liver Diseases. Hepatology.

[2] Abd El-Kader S.M., El-Den Ashmawy E.M. (2015). Non-alcoholic fatty liver disease: The diagnosis and management. World J. Hepatol..

[3] Yki-Jarvinen H. (2014). Non-alcoholic fatty liver disease as a cause and a consequence of metabolic syndrome. Lancet Diabetes Endocrinol..

[4] Younossi Z.M., Koenig A.B., Abdelatif D., Fazel Y., Henry L., Wymer M. (2016). Global epidemiology of nonalcoholic fatty liver disease-Meta-analytic assessment of prevalence, incidence, and outcomes. Hepatology.

[5] Samji N.S., Verma R., Satapathy S.K. (2019). Magnitude of Nonalcoholic Fatty Liver Disease: Western Perspective. J. Clin. Exp. Hepatol..

[6] Tsochatzis E.A., Newsome P.N. (2018). Non-alcoholic fatty liver disease and the interface between primary and secondary care. Lancet Gastroenterol. Hepatol..

[7] Alves-Bezerra M., Cohen D.E. (2017). Triglyceride Metabolism in the Liver. Compr. Physiol..

[8] Kalinowski A., Humphreys K. (2016). Governmental standard drink definitions and low-risk alcohol consumption guidelines in 37 countries. Addiction.

[9] Kanwal F., Kramer J.R., Mapakshi S., Natarajan Y., Chayanupatkul M., Richardson P.A., Li L., Desiderio R., Thrift A.P., Asch S.M. (2018). Risk of Hepatocellular Cancer in Patients with Non-Alcoholic Fatty Liver Disease. Gastroenterology.

[10] Younossi Z., Stepanova M., Ong J.P., Jacobson I.M., Bugianesi E., Duseja A., Eguchi Y., Wong V.W., Negro F., Yilmaz Y. (2019). Nonalcoholic Steatohepatitis Is the Fastest Growing Cause of Hepatocellular Carcinoma in Liver Transplant Candidates. Clin. Gastroenterol. Hepatol..

[11] Vernon G., Baranova A., Younossi Z.M. (2011). Systematic review: The epidemiology and natural history of non-alcoholic fatty liver disease and non-alcoholic steatohepatitis in adults. Aliment. Pharmacol. Ther..

[12] Kabbany M.N., Conjeevaram Selvakumar P.K., Watt K., Lopez R., Akras Z., Zein N., Carey W., Alkhouri N. (2017). Prevalence of Nonalcoholic Steatohepatitis-Associated Cirrhosis in the United States: An Analysis of National Health and Nutrition Examination Survey Data. Am. J. Gastroenterol..

[13] Clifton P. (2019). Metabolic Syndrome-Role of Dietary Fat Type and Quantity. Nutrients.

[14] Kassi E., Pervanidou P., Kaltsas G., Chrousos G. (2011). Metabolic syndrome: Definitions and controversies. BMC Med..

[15] Alberti K.G., Zimmet P., Shaw J., IDF Epidemiology Task Force Consensus Group (2005). The metabolic syndrome—A new worldwide definition. Lancet.

[16] Beltran-Sanchez H., Harhay M.O., Harhay M.M., McElligott S. (2013). Prevalence and trends of metabolic syndrome in the adult U.S. population, 1999–2010. J. Am. Coll. Cardiol..

[17] Younossi Z.M., Golabi P., de Avila L., Paik J.M., Srishord M., Fukui N., Qiu Y., Burns L., Afendy A., Nader F. (2019). The global epidemiology of NAFLD and NASH in patients with type 2 diabetes: A systematic review and meta-analysis. J. Hepatol..

[18] Ortiz-Lopez C., Lomonaco R., Orsak B., Finch J., Chang Z., Kochunov V.G., Hardies J., Cusi K. (2012). Prevalence of prediabetes and diabetes and metabolic profile of patients with nonalcoholic fatty liver disease (NAFLD). Diabetes Care.

[19] Wu S., Wu F., Ding Y., Hou J., Bi J., Zhang Z. (2016). Association of non-alcoholic fatty liver disease with major adverse cardiovascular events: A systematic review and meta-analysis. Sci. Rep..

[20] Younossi Z., Henry L. (2016). Contribution of Alcoholic and Nonalcoholic Fatty Liver Disease to the Burden of Liver-Related Morbidity and Mortality. Gastroenterology.

[21] Kim D., Kim W.R. (2017). Nonobese Fatty Liver Disease. Clin. Gastroenterol. Hepatol..

[22] Benedict M., Zhang X. (2017). Non-alcoholic fatty liver disease: An expanded review. World J. Hepatol..

[23] Caldwell S.H., Chang C.Y., Nakamoto R.K., Krugner-Higby L. (2004). Mitochondria in nonalcoholic fatty liver disease. Clin. Liver Dis..

[24] Donnelly K.L., Smith C.I., Schwarzenberg S.J., Jessurun J., Boldt M.D., Parks E.J. (2005). Sources of fatty acids stored in liver and secreted via lipoproteins in patients with nonalcoholic fatty liver disease. J. Clin. Investig..

[25] Machado M.V., Cortez-Pinto H. (2014). Non-alcoholic fatty liver disease: What the clinician needs to know. World J. Gastroenterol..

[26] Wang D.Q., Portincasa P., Neuschwander-Tetri B.A. (2013). Steatosis in the liver. Compr. Physiol..

[27] Noureddin M., Sanyal A.J. (2018). Pathogenesis of NASH: The Impact of Multiple Pathways. Curr. Hepatol. Rep..

[28] Tilg H., Moschen A.R. (2010). Evolution of inflammation in nonalcoholic fatty liver disease: The multiple parallel hits hypothesis. Hepatology.

[29] Miele L., Valenza V., La Torre G., Montalto M., Cammarota G., Ricci R., Masciana R., Forgione A., Gabrieli M.L., Perotti G. (2009). Increased intestinal permeability and tight junction alterations in nonalcoholic fatty liver disease. Hepatology.

[30] Boursier J., Mueller O., Barret M., Machado M., Fizanne L., Araujo-Perez F., Guy C.D., Seed P.C., Rawls J.F., David L.A. (2016). The severity of nonalcoholic fatty liver disease is associated with gut dysbiosis and shift in the metabolic function of the gut microbiota. Hepatology.

[31] Loomba R., Seguritan V., Li W., Long T., Klitgord N., Bhatt A., Dulai P.S., Caussy C., Bettencourt R., Highlander S.K. (2017). Gut Microbiome-Based Metagenomic Signature for Non-invasive Detection of Advanced Fibrosis in Human Nonalcoholic Fatty Liver Disease. Cell Metab..

[32] Luck H., Tsai S., Chung J., Clemente-Casares X., Ghazarian M., Revelo X.S., Lei H., Luk C.T., Shi S.Y., Surendra A. (2015). Regulation of obesity-related insulin resistance with gut anti-inflammatory agents. Cell Metab..

[33] Albillos A., Gottardi A., Rescigno M. (2019). The gut-liver axis in liver disease: Pathophysiological basis for therapy. J. Hepatol..

[34] Iqbal U., Perumpail B.J., Akhtar D., Kim D., Ahmed A. (2019). The Epidemiology, Risk Profiling and Diagnostic Challenges of Nonalcoholic Fatty Liver Disease. Medicines.

[35] Younossi Z., Anstee Q.M., Marietti M., Hardy T., Henry L., Eslam M., George J., Bugianesi E. (2018). Global burden of NAFLD and NASH: Trends, predictions, risk factors and prevention. Nat. Rev. Gastroenterol. Hepatol..

[36] Yang J.D., Abdelmalek M.F., Pang H., Guy C.D., Smith A.D., Diehl A.M., Suzuki A. (2014). Gender and menopause impact severity of fibrosis among patients with nonalcoholic steatohepatitis. Hepatology.

[37] Valenti L.V.C., Baselli G.A. (2018). Genetics of Nonalcoholic Fatty Liver Disease: A 2018 Update. Curr. Pharm. Des..

[38] Romeo S., Kozlitina J., Xing C., Pertsemlidis A., Cox D., Pennacchio L.A., Boerwinkle E., Cohen J.C., Hobbs H.H. (2008). Genetic variation in PNPLA3 confers susceptibility to nonalcoholic fatty liver disease. Nat. Genet..

[39] Burza M.A., Pirazzi C., Maglio C., Sjoholm K., Mancina R.M., Svensson P.A., Jacobson P., Adiels M., Baroni M.G., Boren J. (2012). PNPLA3 I148M (rs738409) genetic variant is associated with hepatocellular carcinoma in obese individuals. Dig. Liver Dis..

[40] Pingitore P., Romeo S. (2019). The role of PNPLA3 in health and disease. Biochim. Biophys. Acta Mol. Cell Biol. Lipids.

[41] Lazo M., Hernaez R., Eberhardt M.S., Bonekamp S., Kamel I., Guallar E., Koteish A., Brancati F.L., Clark J.M. (2013). Prevalence of nonalcoholic fatty liver disease in the United States: The Third National Health and Nutrition Examination Survey, 1988–1994. Am. J. Epidemiol..

[42] Jensen M.D., Ryan D.H., Apovian C.M., Ard J.D., Comuzzie A.G., Donato K.A., Hu F.B., Hubbard V.S., Jakicic J.M., Kushner R.F. (2014). 2013 AHA/ACC/TOS guideline for the management of overweight and obesity in adults: A report of the American College of Cardiology/American Heart Association Task Force on Practice Guidelines and The Obesity Society. Circulation.

[43] Wagner A., Dallongeville J., Haas B., Ruidavets J.B., Amouyel P., Ferrieres J., Simon C., Arveiler D. (2012). Sedentary behaviour, physical activity and dietary patterns are independently associated with the metabolic syndrome. Diabetes Metab..

[44] Hamilton M.T., Hamilton D.G., Zderic T.W. (2007). Role of low energy expenditure and sitting in obesity, metabolic syndrome, type 2 diabetes, and cardiovascular disease. Diabetes.

[45] Vallance J.K., Gardiner P.A., Lynch B.M., D′Silva A., Boyle T., Taylor L.M., Johnson S.T., Buman M.P., Owen N. (2018). Evaluating the Evidence on Sitting, Smoking, and Health: Is Sitting Really the New Smoking?. Am. J. Public Health.

[46] Dunstan D.W., Salmon J., Healy G.N., Shaw J.E., Jolley D., Zimmet P.Z., Owen N., AusDiab Steering C. (2007). Association of television viewing with fasting and 2-h postchallenge plasma glucose levels in adults without diagnosed diabetes. Diabetes Care.

[47] Healy G.N., Dunstan D.W., Salmon J., Shaw J.E., Zimmet P.Z., Owen N. (2008). Television time and continuous metabolic risk in physically active adults. Med. Sci. Sports Exerc..

[48] Snowling N.J., Hopkins W.G. (2006). Effects of different modes of exercise training on glucose control and risk factors for complications in type 2 diabetic patients: A meta-analysis. Diabetes Care.

[49] Perseghin G., Lattuada G., De Cobelli F., Ragogna F., Ntali G., Esposito A., Belloni E., Canu T., Terruzzi I., Scifo P. (2007). Habitual physical activity is associated with intrahepatic fat content in humans. Diabetes Care.

[50] Ryu S., Chang Y., Jung H.S., Yun K.E., Kwon M.J., Choi Y., Kim C.W., Cho J., Suh B.S., Cho Y.K. (2015). Relationship of sitting time and physical activity with non-alcoholic fatty liver disease. J. Hepatol..

[51] Assy N., Nasser G., Kamayse I., Nseir W., Beniashvili Z., Djibre A., Grosovski M. (2008). Soft drink consumption linked with fatty liver in the absence of traditional risk factors. Can. J. Gastroenterol..

[52] Hosseini Z., Whiting S.J., Vatanparast H. (2016). Current evidence on the association of the metabolic syndrome and dietary patterns in a global perspective. Nutr. Res. Rev..

[53] Stephenson K., Kennedy L., Hargrove L., Demieville J., Thomson J., Alpini G., Francis H. (2018). Updates on Dietary Models of Nonalcoholic Fatty Liver Disease: Current Studies and Insights. Gene Expr..

[54] Oddy W.H., Herbison C.E., Jacoby P., Ambrosini G.L., O’Sullivan T.A., Ayonrinde O.T., Olynyk J.K., Black L.J., Beilin L.J., Mori T.A. (2013). The Western dietary pattern is prospectively associated with nonalcoholic fatty liver disease in adolescence. Am. J. Gastroenterol..

[55] Kechagias S., Ernersson A., Dahlqvist O., Lundberg P., Lindstrom T., Nystrom F.H., Fast Food Study Group (2008). Fast-food-based hyper-alimentation can induce rapid and profound elevation of serum alanine aminotransferase in healthy subjects. Gut.

[56] Yasutake K., Kohjima M., Kotoh K., Nakashima M., Nakamuta M., Enjoji M. (2014). Dietary habits and behaviors associated with nonalcoholic fatty liver disease. World J. Gastroenterol..

[57] Nishi T., Babazono A., Maeda T., Imatoh T., Une H. (2016). Effects of Eating Fast and Eating Before Bedtime on the Development of Nonalcoholic Fatty Liver Disease. Popul. Health Manag..

[58] Koopman K.E., Caan M.W., Nederveen A.J., Pels A., Ackermans M.T., Fliers E., la Fleur S.E., Serlie M.J. (2014). Hypercaloric diets with increased meal frequency, but not meal size, increase intrahepatic triglycerides: A randomized controlled trial. Hepatology.

[59] Leoni S., Tovoli F., Napoli L., Serio I., Ferri S., Bolondi L. (2018). Current guidelines for the management of non-alcoholic fatty liver disease: A systematic review with comparative analysis. World J. Gastroenterol..

[60] Pallayova M., Taheri S. (2014). Non-alcoholic fatty liver disease in obese adults: Clinical aspects and current management strategies. Clin. Obes..

[61] Palmer M., Schaffner F. (1990). Effect of weight reduction on hepatic abnormalities in overweight patients. Gastroenterology.

[62] Kugelmas M., Hill D.B., Vivian B., Marsano L., McClain C.J. (2003). Cytokines and NASH: A pilot study of the effects of lifestyle modification and vitamin E. Hepatology.

[63] Hickman I.J., Jonsson J.R., Prins J.B., Ash S., Purdie D.M., Clouston A.D., Powell E.E. (2004). Modest weight loss and physical activity in overweight patients with chronic liver disease results in sustained improvements in alanine aminotransferase, fasting insulin, and quality of life. Gut.

[64] Tamura Y., Tanaka Y., Sato F., Choi J.B., Watada H., Niwa M., Kinoshita J., Ooka A., Kumashiro N., Igarashi Y. (2005). Effects of diet and exercise on muscle and liver intracellular lipid contents and insulin sensitivity in type 2 diabetic patients. J. Clin. Endocrinol. Metab..

[65] Andersen T., Gluud C., Franzmann M.B., Christoffersen P. (1991). Hepatic effects of dietary weight loss in morbidly obese subjects. J. Hepatol..

[66] Koutoukidis D.A., Astbury N.M., Tudor K.E., Morris E., Henry J.A., Noreik M., Jebb S.A., Aveyard P. (2019). Association of Weight Loss Interventions with Changes in Biomarkers of Nonalcoholic Fatty Liver Disease: A Systematic Review and Meta-analysis. JAMA Intern. Med..

[67] Wong V.W., Chan R.S., Wong G.L., Cheung B.H., Chu W.C., Yeung D.K., Chim A.M., Lai J.W., Li L.S., Sea M.M. (2013). Community-based lifestyle modification programme for non-alcoholic fatty liver disease: A randomized controlled trial. J. Hepatol..

[68] Harrison S.A., Fecht W., Brunt E.M., Neuschwander-Tetri B.A. (2009). Orlistat for overweight subjects with nonalcoholic steatohepatitis: A randomized, prospective trial. Hepatology.

[69] Vilar-Gomez E., Martinez-Perez Y., Calzadilla-Bertot L., Torres-Gonzalez A., Gra-Oramas B., Gonzalez-Fabian L., Friedman S.L., Diago M., Romero-Gomez M. (2015). Weight Loss Through Lifestyle Modification Significantly Reduces Features of Nonalcoholic Steatohepatitis. Gastroenterology.

[70] Kwak M.S., Kim D. (2018). Non-alcoholic fatty liver disease and lifestyle modifications, focusing on physical activity. Korean J. Intern. Med..

[71] Sargeant J.A., Gray L.J., Bodicoat D.H., Willis S.A., Stensel D.J., Nimmo M.A., Aithal G.P., King J.A. (2018). The effect of exercise training on intrahepatic triglyceride and hepatic insulin sensitivity: A systematic review and meta-analysis. Obes. Rev..

[72] European Association for the Study of the Liver (EASL), European Association for the Study of Disbetes (EASD), European Association for the Study of Obesity (EASO) (2016). EASL-EASD-EASO Clinical Practice Guidelines for the management of non-alcoholic fatty liver disease. J. Hepatol..

[73] Orci L.A., Gariani K., Oldani G., Delaune V., Morel P., Toso C. (2016). Exercise-based Interventions for Nonalcoholic Fatty Liver Disease: A Meta-analysis and Meta-regression. Clin. Gastroenterol. Hepatol..

[74] van der Heijden G.J., Wang Z.J., Chu Z.D., Sauer P.J., Haymond M.W., Rodriguez L.M., Sunehag A.L. (2010). A 12-week aerobic exercise program reduces hepatic fat accumulation and insulin resistance in obese, Hispanic adolescents. Obesity (Silver Spring).

[75] Hashida R., Kawaguchi T., Bekki M., Omoto M., Matsuse H., Nago T., Takano Y., Ueno T., Koga H., George J. (2017). Aerobic vs. resistance exercise in non-alcoholic fatty liver disease: A systematic review. J. Hepatol..

[76] Younossi Z.M., Loomba R., Anstee Q.M., Rinella M.E., Bugianesi E., Marchesini G., Neuschwander-Tetri B.A., Serfaty L., Negro F., Caldwell S.H. (2018). Diagnostic modalities for nonalcoholic fatty liver disease, nonalcoholic steatohepatitis, and associated fibrosis. Hepatology.

[77] Grundy S.M., Abate N., Chandalia M. (2002). Diet composition and the metabolic syndrome: What is the optimal fat intake?. Am. J. Med..

[78] York L.W., Puthalapattu S., Wu G.Y. (2009). Nonalcoholic fatty liver disease and low-carbohydrate diets. Annu. Rev. Nutr..

[79] Sharman M.J., Kraemer W.J., Love D.M., Avery N.G., Gomez A.L., Scheett T.P., Volek J.S. (2002). A ketogenic diet favorably affects serum biomarkers for cardiovascular disease in normal-weight men. J. Nutr..

[80] Volek J., Sharman M., Gomez A., Judelson D., Rubin M., Watson G., Sokmen B., Silvestre R., French D., Kraemer W. (2004). Comparison of energy-restricted very low-carbohydrate and low-fat diets on weight loss and body composition in overweight men and women. Nutr. Metab. (Lond.).

[81] Foster G.D., Wyatt H.R., Hill J.O., McGuckin B.G., Brill C., Mohammed B.S., Szapary P.O., Rader D.J., Edman J.S., Klein S. (2003). A randomized trial of a low-carbohydrate diet for obesity. N. Engl. J. Med..

[82] Asrih M., Jornayvaz F.R. (2014). Diets and nonalcoholic fatty liver disease: The good and the bad. Clin. Nutr..

[83] Eckel R.H., Jakicic J.M., Ard J.D., de Jesus J.M., Houston Miller N., Hubbard V.S., Lee I.M., Lichtenstein A.H., Loria C.M., Millen B.E. (2014). 2013 AHA/ACC guideline on lifestyle management to reduce cardiovascular risk: A report of the American College of Cardiology/American Heart Association Task Force on Practice Guidelines. Circulation.

[84] Properzi C., O′Sullivan T.A., Sherriff J.L., Ching H.L., Jeffrey G.P., Buckley R.F., Tibballs J., MacQuillan G.C., Garas G., Adams L.A. (2018). Ad Libitum Mediterranean and Low-Fat Diets Both Significantly Reduce Hepatic Steatosis: A Randomized Controlled Trial. Hepatology.

[85] Schwartz M.W., Seeley R.J., Zeltser L.M., Drewnowski A., Ravussin E., Redman L.M., Leibel R.L. (2017). Obesity Pathogenesis: An Endocrine Society Scientific Statement. Endocr. Rev..

[86] Sacks F.M., Bray G.A., Carey V.J., Smith S.R., Ryan D.H., Anton S.D., McManus K., Champagne C.M., Bishop L.M., Laranjo N. (2009). Comparison of weight-loss diets with different compositions of fat, protein, and carbohydrates. N. Engl. J. Med..

[87] Jang E.C., Jun D.W., Lee S.M., Cho Y.K., Ahn S.B. (2018). Comparison of efficacy of low-carbohydrate and low-fat diet education programs in non-alcoholic fatty liver disease: A randomized controlled study. Hepatol. Res..

[88] Chiavaroli L., Viguiliouk E., Nishi S.K., Blanco Mejia S., Rahelic D., Kahleova H., Salas-Salvado J., Kendall C.W., Sievenpiper J.L. (2019). DASH Dietary Pattern and Cardiometabolic Outcomes: An Umbrella Review of Systematic Reviews and Meta-Analyses. Nutrients.

[89] Razavi Zade M., Telkabadi M.H., Bahmani F., Salehi B., Farshbaf S., Asemi Z. (2016). The effects of DASH diet on weight loss and metabolic status in adults with non-alcoholic fatty liver disease: A randomized clinical trial. Liver Int..

[90] Xiao M.L., Lin J.S., Li Y.H., Liu M., Deng Y.Y., Wang C.Y., Chen Y.M. (2019). Adherence to the Dietary Approaches to Stop Hypertension (DASH) diet is associated with lower presence of non-alcoholic fatty liver disease in middle-aged and elderly adults. Public Health Nutr..

[91] Miller E.R., Erlinger T.P., Appel L.J. (2006). The effects of macronutrients on blood pressure and lipids: An overview of the DASH and OmniHeart trials. Curr. Atheroscler. Rep..

[92] Bugianesi E. (2016). EASL-EASD-EASO Clinical Practice Guidelines for the management of non-alcoholic fatty liver disease: Disease mongering or call to action?. Diabetologia.

[93] de la Iglesia R., Loria-Kohen V., Zulet M.A., Martinez J.A., Reglero G., Ramirez de Molina A. (2016). Dietary Strategies Implicated in the Prevention and Treatment of Metabolic Syndrome. Int. J. Mol. Sci..

[94] Kaliora A.C., Gioxari A., Kalafati I.P., Diolintzi A., Kokkinos A., Dedoussis G.V. (2019). The Effectiveness of Mediterranean Diet in Nonalcoholic Fatty Liver Disease Clinical Course: An Intervention Study. J. Med. Food.

[95] Gepner Y., Shelef I., Komy O., Cohen N., Schwarzfuchs D., Bril N., Rein M., Serfaty D., Kenigsbuch S., Zelicha H. (2019). The beneficial effects of Mediterranean diet over low-fat diet may be mediated by decreasing hepatic fat content. J. Hepatol..

[96] Fakhry T.K., Mhaskar R., Schwitalla T., Muradova E., Gonzalvo J.P., Murr M.M. (2019). Bariatric surgery improves nonalcoholic fatty liver disease: A contemporary systematic review and meta-analysis. Surg. Obes. Relat. Dis..

[97] Wang Y.W., He S.J., Feng X., Cheng J., Luo Y.T., Tian L., Huang Q. (2017). Metformin: A review of its potential indications. Drug Des. Dev. Ther..

[98] Said A., Akhter A. (2017). Meta-Analysis of Randomized Controlled Trials of Pharmacologic Agents in Non-alcoholic Steatohepatitis. Ann. Hepatol..

[99] Gruman J., Rovner M.H., French M.E., Jeffress D., Sofaer S., Shaller D., Prager D.J. (2010). From patient education to patient engagement: Implications for the field of patient education. Patient Educ. Couns..

[100] Murali N.S., Deao C.E. (2019). Patient Engagement. Prim. Care.

[101] Hamman R.F., Wing R.R., Edelstein S.L., Lachin J.M., Bray G.A., Delahanty L., Hoskin M., Kriska A.M., Mayer-Davis E.J., Pi-Sunyer X. (2006). Effect of weight loss with lifestyle intervention on risk of diabetes. Diabetes Care.

[102] Glen J., Floros L., Day C., Pryke R., Guideline Development Group (2016). Non-alcoholic fatty liver disease (NAFLD): Summary of NICE guidance. BMJ.

[103] Moolla A., Motohashi K., Marjot T., Shard A., Ainsworth M., Gray A., Holman R., Pavlides M., Ryan J.D., Tomlinson J.W. (2019). A multidisciplinary approach to the management of NAFLD is associated with improvement in markers of liver and cardio-metabolic health. Frontline Gastroenterol..

[104] Cobbold J.F.L., Raveendran S., Peake C.M., Anstee Q.M., Yee M.S., Thursz M.R. (2013). Piloting a multidisciplinary clinic for the management of non-alcoholic fatty liver disease: Initial 5-year experience. Frontline Gastroenterol..

[105] Estruch R., Ros E., Salas-Salvado J., Covas M.I., Corella D., Aros F., Gomez-Gracia E., Ruiz-Gutierrez V., Fiol M., Lapetra J. (2013). Primary prevention of cardiovascular disease with a Mediterranean diet. N. Engl. J. Med..

[106] Liu R.H. (2013). Dietary bioactive compounds and their health implications. J. Food Sci..

[107] Saha P., Talukdar A.D., Nath R., Sarker S.D., Nahar L., Sahu J., Choudhury M.D. (2019). Role of Natural Phenolics in Hepatoprotection: A Mechanistic Review and Analysis of Regulatory Network of Associated Genes. Front. Pharmacol..

[108] Zelber-Sagi S., Salomone F., Mlynarsky L. (2017). The Mediterranean dietary pattern as the diet of choice for non-alcoholic fatty liver disease: Evidence and plausible mechanisms. Liver Int..

[109] Jensen T., Abdelmalek M.F., Sullivan S., Nadeau K.J., Green M., Roncal C., Nakagawa T., Kuwabara M., Sato Y., Kang D.H. (2018). Fructose and sugar: A major mediator of non-alcoholic fatty liver disease. J. Hepatol..

[110] Abenavoli L., Milanovic M., Milic N., Luzza F., Giuffre A.M. (2019). Olive oil antioxidants and non-alcoholic fatty liver disease. Expert Rev. Gastroenterol. Hepatol..

[111] Barrera C., Valenzuela R., Rincon M.A., Espinosa A., Echeverria F., Romero N., Gonzalez-Manan D., Videla L.A. (2018). Molecular mechanisms related to the hepatoprotective effects of antioxidant-rich extra virgin olive oil supplementation in rats subjected to short-term iron administration. Free Radic. Biol. Med..

[112] Soto-Alarcon S.A., Valenzuela R., Valenzuela A., Videla L.A. (2018). Liver Protective Effects of Extra Virgin Olive Oil: Interaction between Its Chemical Composition and the Cell-signaling Pathways Involved in Protection. Endocr. Metab. Immune Disord. Drug Targets.

[113] Godos J., Federico A., Dallio M., Scazzina F. (2017). Mediterranean diet and nonalcoholic fatty liver disease: Molecular mechanisms of protection. Int. J. Food Sci. Nutr..

[114] Mirmiran P., Gaeini Z., Bahadoran Z., Azizi F. (2019). Elevated serum levels of aminotransferases in relation to unhealthy foods intake: Tehran lipid and glucose study. BMC Endocr. Disord..

[115] Borg G.A. (1982). Psychophysical bases of perceived exertion. Med. Sci. Sports Exerc..

[116] Martin-Borras C., Gine-Garriga M., Puig-Ribera A., Martin C., Sola M., Cuesta-Vargas A.I., PPAF Group (2018). A new model of exercise referral scheme in primary care: Is the effect on adherence to physical activity sustainable in the long term? A 15-month randomised controlled trial. BMJ Open.

